# Aligning Patient Discharge Curricula to Patient-Reported Outcomes in Sub Internship

**DOI:** 10.1007/s11606-026-10495-3

**Published:** 2026-05-11

**Authors:** Radhika Sreedhar, Rachel Yudkowsky, Asra Khan, Ananya Gangopadhyaya, Angie Fanuke, Jon Radosta, Yoon Soo Park

**Affiliations:** 1 Department of Medicine, University of Illinois College of Medicine, Chicago, IL USA; 2 Department of Medical Education, University of Illinois College of Medicine, Chicago, IL USA

**Keywords:** patient discharge curricula, medical student, workplace-based observation

## Abstract

**Background:**

Patients’ lack of understanding of post-discharge treatment plans can result in readmissions and adverse events after discharge from the hospital. Despite this, there are few structured curricula for medical students to learn about safely discharging patients.

Objective: To determine the ability of a structured curriculum to improve medical students’ knowledge and skills about discharge instructions.

**Methods:**

Design and participants: This is a mixed-methods study involving fourth-year medical students in their internal medicine sub-internship.

Interventions: The patient discharge curriculum consisted of (a) self-study of patient discharge didactics, (b) direct observation and feedback of discharge instructions provided by students, (c) student-initiated post-discharge phone call to patients, and (d) a small group debrief activity.

Main measures: Skills were assessed using a standardized workplace-based direct observation of students providing discharge instructions. Changes in knowledge and skills were measured by differences in pre- and post-curriculum survey scores using a paired *t* test. Logistic regression was used to determine if the scores on direct observation could predict patient-reported outcomes.

**Key Results:**

All self-reported knowledge and skills outcomes related to patient discharges improved significantly after the curriculum (*p* < 0.001). The direct observation of students providing discharge instructions to patients after completing the curriculum revealed that 75% of students were able to independently perform all the discharge-related tasks. There was a significant association between scores on the direct observation of students and patient-reported knowledge of what to do in urgent or emergent situations (OR = 29.52, *p* = 0.002). Based on student feedback, follow-up phone calls to patients were a powerful motivator for them to improve discharge instructions.

**Conclusions:**

This study offers valuable evidence supporting the use of a structured discharge curriculum. This structured training in providing discharge instructions improves medical students’ knowledge and skills about discharge instructions and positively impacts patient-reported outcomes.

**Supplementary Information:**

The online version contains supplementary material available at 10.1007/s11606-026-10495-3.

## INTRODUCTION

### Background

Patients’ lack of understanding of the post-discharge treatment plan can result in readmissions and adverse events following their discharge from the hospital.^1,2^ Based on the Agency for Healthcare Research and Quality (AHRQ) data for 2020, there were 14 readmissions for every 100 adult admissions, with an average cost of $17,700 per readmission.^3^ In a study of clinical pharmacist interventions for patients on high-risk medications, 27% of patients experienced an adverse event and 18% had clinically important medication errors following hospital discharge.^4^

Studies show that when there are systems in place to arrange follow-up appointments, confirm medication reconciliation, and conduct individual patient education, readmissions can be reduced by 30%.^5^

The frequency of errors related to patient discharges is exacerbated when patient discharges are completed by first-year residents who have not had the necessary training as medical students to handle the complexities of discharging patients. A study in which patient discharges by residents were directly observed by trained observers revealed that close to half of the patients were not told the reason for follow-up appointments, 81% of patients did not receive guidance on red-flag signs, and only one patient was asked to teach back his understanding of the discharge plan.^6^ In a study by Trivedi et al., only 50% of the residents indicated that they received formal instruction on discharging patients.^7^ The same study also revealed that experiential learning from patients post-discharge is a motivator to change discharge practices. A survey by Carnahan et al. revealed that over 50% of residents felt that their education about discharge was sub-optimal.^8^ A survey of emergency medicine residency programs revealed that fewer than half the programs provide formal training about discharge to interns at orientation.^9^

Most studies on discharge curricula for medical students assessed knowledge, skills, and attitudes before and after implementation of a curriculum.^10–14^ Lyons et al.^15^ created a discharge workshop and direct observation tool to provide feedback for students, and a study of medical students by Eden et al. incorporated the use of a checklist for discharging patients.^16^

While workplace-based assessments (WBA) can provide opportunities for immediate performance feedback to guide learning,^17^ there are few structured curricula using standardized workplace-based assessment tools^18,19^ for assessing medical students’ ability to provide discharge instructions to patients. There are little data relating discharge curricula or WBA to patient-reported outcomes.

### Objective

The objective of this study was to evaluate whether a structured curriculum would improve students’ ability to provide verbal discharge instructions to patients. We hypothesized that student's exposure to a structured discharge curriculum would improve their knowledge and skills to provide discharge instructions to patients.

## METHODS

### Design, Participants, and Setting

This is a mixed-method study with an observational component consisting of a cross-sectional survey and a qualitative evaluation of students’ comments. Study participants were fourth-year medical students on an inpatient internal medicine sub-internship from 2020 to 2024. No visiting fourth-year students from other institutions were included in the study. The study was conducted at the academic teaching hospital of the University of Illinois College of Medicine (UICOM) Chicago campus and related community hospital sites.

### Interventions

The patient discharge curriculum was organized into three components: (1) self-study of didactics related to discharge, (2) experiential with (a) direct observation of discharge instructions provided by students with feedback and (b) student-led post-discharge phone call to patients to assess for problems related to discharge at the UICOM site, (3) preceptor-led small group debrief activity of discharges.

In the “self-study” component, completed in the first week of the sub-internship, learners (a) completed a self-assessment of their knowledge, attitudes, and skills regarding discharging patients using an Entrustable Professional Activity (EPA)-aligned pre-curriculum survey (Appendix 5); (b) reviewed video recordings of good and bad discharges; and (c) reflected on the videos regarding optimal discharge practices.

In the “experiential” component, students worked with multidisciplinary staff, faculty, and residents to perform all the tasks related to patient discharge including providing verbal discharge instructions. Skill acquisition for discharging patients was accomplished by students practicing various discharge components when their patients were being discharged. All students were required to (a) discharge at least two patients during the rotation and (b) have at least one direct observation of discharge as a part of the sub-internship. Students received direct feedback on providing discharge instructions using the EPA-aligned Discharge Observation Tool (DOT). The feedback was provided by the residents on the team who knew the patient immediately after completion of the exercise. All residents were informed of their responsibilities towards medical student education at the beginning of the academic year. At the start of the sub-internship, students were instructed to submit two completed DOTs, and were responsible for reminding residents to observe them providing the discharge instructions to their patients.

Students assigned to the academic teaching hospital at UICOM-Chicago were also required to complete at least two post-discharge phone calls to their patients within a week of discharge. The purpose of the call was to assess the adequacy of discharge instructions and determine if there were any problems related to the discharge process. Students used a modified template created by the AHRQ to guide their phone calls (Appendix 1). They completed self-reflections on areas of improvement of discharge instructions based on patient feedback. They had to turn in the post-discharge phone call documentation, which listed patients’ understanding of their admitting diagnosis, alarm symptoms, medications, follow-up appointments, and problems patients experienced after discharge. Students also indicated how they resolved these problems and provided their reflections on areas of improvement.

The preceptor-led small group activity was a 1-h interactive session that addressed common problems related to discharge. This session was usually led by the first author in the first 2 weeks of the rotation so students could share their experiences and make changes. The preceptor led a discussion about issues uncovered by students related to discharge from follow-up phone calls, and the group brainstormed ways to prevent errors and improve the safety of hospital discharge. This was followed by a discussion about error reporting and the principles of quality improvement to help improve the quality of discharges. Students completed a post-curriculum survey (Appendix 6) reflecting on lessons learned and planned changes at the rotation’s end.

### Study Instrument

A workplace-based DOT (Appendix 2) was used to assess the performance of tasks essential to safe and effective discharge communication. A consensus panel created the tool after several rounds of deliberation, pilot testing, and modification. The panel consisted of three faculty members board certified in internal medicine, one chief resident, and one third-year medical student. Items included in the DOT were based on best practices found in the literature on patient discharge.^20–22^ Each item was scored on a 5-point modified Ottawa scale, ^23^ with each scale point anchored by phrases describing the student’s ability to do the task with varying degrees of independence. Overall DOT scores were determined by taking the mean across DOT items. An internal validation study was done to determine content validity, which showed that all items were rated as relevant (Appendix 3). The consistency between trained raters using this form was 0.8 as measured by intraclass correlation (ICC), 95% CI (0.59–0.96) (Appendix 4).

### Primary Outcomes

Students’ skills in providing discharge instructions to patients were assessed by residents using the DOT. The students’ mean score for each domain and percentage of students able to do the task independently were reported.

### Secondary Outcomes

Self-reported knowledge and skills regarding discharge was assessed using changes in scores from the pre- to post-curriculum surveys.

Associations between overall DOT scores and six patient-reported outcomes: (1) knowing their diagnosis, (2) knowing whether they have a follow-up appointment, (3) knowing whether they have a specialist appointment confirmed, (4) identifying potential barriers to attendance, (5) potential solution identified, and (6) knowing what to do in urgent situations was also studied.

### Statistical Analysis

Data analyses were conducted using Stata 18 MP (College Station, TX).^24^ Paired *t* test was used to compare means of continuous variables in the pre- and post-curriculum survey. Logistic regression analyses were used to determine the associations between overall DOT scores and patient-reported outcomes.

### Ethical Considerations

The Institutional Review Board of the University of Illinois at Chicago approved this study as exempt.

## KEY RESULTS

Four cohorts of fourth-year students (*n* = 386) doing a sub-internship in internal medicine were assessed using the DOT between August 17, 2020, and April 24, 2024. The DOT was used to provide real-time feedback on discharge tasks for 377 students.

### Performance on DOT

Seventy-five percent of students were able to do all the tasks independently. The ability to answer patients’ questions was the item which had the highest percentage of students reporting as being able to perform the task independently. Students’ ability to assess if the patients could carry out the post-discharge plan (cost, transportation, insurance, etc.) was the area where only 75% were rated as being able to independently perform the task. The mean scores for the items were similar across the four cohorts of students. Student performance is shown in Table 1.

**Table 1:** Student Performance in Workplace-Based Observation of Discharge Instructions

	Students able to do task independently *n* (%)	Mean ± std. dev of overall score AY20–24 (*n* = 377)
1. Patient education		
a. The student was able to explain major diagnosis and hospital course	327/377 (87)	4.86 ± 0.36
b. The student was able to explain alarm symptoms or problems requiring return to ER or call to PCP	332/377 (88)	4.87 ± 0.37
2. Patient-centered care		
a. The student was able to elicit concerns about discharge and home support	318/377 (84)	4.83 ± 0.41
b. The student was able to assess patients’ ability to carry out plan (cost, transportation, insurance, etc.)	282/375 (75)	4.72 ± 0.52
c. The student was able to address the patient’s concerns and questions	332/377 (88)	4.87 ± 0.38
d. The student was able to notify the patient/family of communication with other providers	339/376 (90)	4.90 ± 0.32
3. Medication reconciliation		
a. The student was able to assess the patient’s safety to take meds (eyesight, dexterity, health literacy, cognitive ability)	293/374 (78)	4.76 ± 0.47
b. The student was able to explain and provide a list of meds to be continued and meds to be STOPPED	326/374 (87)	4.86 ± 0.38
c. The student was able to explain the indication, instructions, and major side effects of all new medications	295/375 (79)	4.76 ± 0.49
4. System-based practice		
a. The student was able to involve team members (CM, SW, PT, etc.) in the discharge^a^	261/315 (83)	4.80 ± 0.46
b. The student was able to communicate a follow-up plan with other MDs	297/371 (80)	4.78 ± 0.47
c. The student was able to explain the timeframe of future tests that require follow-up (i.e., labs, etc.), including which clinician will follow	309/376 (82)	4.81 ± 0.42
5. Communication skills		
a. The student was able to use appropriate language and terminology (avoidance of medical jargon)	342/376 (91)	4.90 ± 0.32
b. The student was able to use the teach-back method during the discussion with the patient	325/374 (87)	4.85 ± 0.42
c. The student was able to answer questions appropriately, demonstrate professionalism/humanism, and use appropriate non-verbal communication	348/376 (93)	4.92 ± 0.29

^a^This item was reported as not being directly observed in 31 students

### Student and Observer Feedback on DOT

Ninety percent of observers and 80% of students agreed or strongly agreed that this workplace-based assessment of students’ ability to provide discharge instructions was a helpful educational experience.

### Changes from Pre- to Post-Curriculum Survey

All 386 students completed the discharge curriculum survey at the end of their internal medicine sub-internship. Ninety-four percent (362/386) of students reported that they provided discharge education to patients most of the time or all the time during the sub-internship. The differences in mean scores from pre- to post-curriculum survey are shown in Table 2.

**Table 2:** Differences in Student Self-reported Mean Scores in Awareness and Knowledge in Various Domains Related to Discharge on Pre- and Post-Curriculum Surveys

Domain	Pre-mean (SD)	Post mean (SD)	Mean difference (post–pre)	95% CI	*p*-value	Number
Awareness of housing instability	3.77 (0.91)	4.47 (0.61)	+ 0.70	0.61 to 0.79	< 0.001	385
Awareness of food insecurity	3.43 (1.01)	4.09 (0.81)	+ 0.67	0.57 to 0.77	< 0.001	385
Awareness of access to utilities	3.20 (1.04)	3.99 (0.89)	+ 0.79	0.67 to 0.90	< 0.001	385
Awareness of transportation needs	3.92 (0.90)	4.65 (0.59)	+ 0.73	0.64 to 0.83	< 0.001	385
Awareness of interpersonal safety	3.42 (1.00)	4.17 (0.81)	+ 0.75	0.64 to 0.86	< 0.001	384
Knowledge: medication reconciliation	2.95 (1.08)	4.16 (0.80)	+ 1.22	1.10 to 1.34	< 0.001	385
Knowledge: writing a discharge summary	3.68 (0.97)	4.59 (0.78)	+ 0.92	0.80 to 1.03	< 0.001	385
Knowledge: roles in discharge planning	3.15 (0.99)	4.06 (0.84)	+ 0.91	0.80 to 1.03	< 0.001	385

Eighty-nine percent (345/386) of students reported that they agreed or strongly agreed that they knew how to do a medication reconciliation, and this was the area that showed the greatest improvement in scores. Ninety-five percent (365/386) of students agreed or strongly agreed with the statement that they know how to write a discharge summary while 84% of students agreed or strongly agreed that they knew the roles of a social worker, discharge planner, and case manager.

Differences in scores from pre- to post-curriculum survey on self-reported students’ ability to consider various aspects related to discharge and answer patient’s questions related to them are shown in Table 3.

**Table 3:** Differences in Student Self-reported Mean Scores in Considering Various Domains in Planning Discharge and Ability to Answer Patient Questions on Pre- and Post-Curriculum Surveys

Measure	Pre-mean (SD)	Post mean (SD)	Mean difference (post–pre)	95% CI	*p*-value	Number
Consider transportation needs	2.18 (0.81)	2.80 (0.45)	+ 0.63	0.54 to 0.71	< 0.001	382
Consider insurance needs	1.64 (0.74)	2.23 (0.79)	+ 0.59	0.50 to 0.68	< 0.001	383
Consider financial concerns	1.83 (0.77)	2.48 (0.67)	+ 0.65	0.57 to 0.73	< 0.001	384
Consider home needs	2.24 (0.75)	2.79 (0.47)	+ 0.55	0.47 to 0.63	< 0.001	384
Answer questions: housing instability	2.37 (0.93)	3.44 (0.83)	+ 1.07	0.97 to 1.16	< 0.001	383
Answer questions: food insecurity	2.42 (0.94)	3.32 (0.92)	+ 0.90	0.80 to 1.00	< 0.001	384
Answer questions: access to utilities	2.10 (0.92)	3.09 (0.96)	+ 0.99	0.89 to 1.09	< 0.001	381
Answer questions: transportation resources	2.69 (0.97)	3.80 (0.80)	+ 1.11	1.02 to 1.21	< 0.001	381
Answer questions: interpersonal safety	2.45 (0.94)	3.51 (0.89)	+ 1.06	0.96 to 1.16	< 0.001	379

Ninety-eight percent (380/386) of students stated that they consider transportation needs most of the time or all the time when discharging a patient. Seventy percent (271/386) of students stated that they needed assistance only in complex situations or did not need assistance when addressing patients’ concerns and questions about access to transportation.

Ninety-eight percent (379/386) of students stated that they consider home needs (O2, RN, PT, etc.) most of the time or all the time during the discharge process. Seventy-eight percent (303/386) of students stated they considered the patients’ insurance, and 91% (351/386) of students stated that they took into account financial concerns most or all the time during the discharge process.

At the end of the sub-internship, the areas of the discharge process that most students felt they would personally work on from a patient safety standpoint were medication reconciliation, patient education, and accurate documentation. The most common areas students reported that they would like to learn more about included the roles of the discharge planner and social worker, levels of care post-discharge, transportation options, medication reconciliation, and social determinants of health.

### Post-discharge Phone calls

Post-discharge phone calls were made by students to the patients they discharged from the university hospital. A total of 428 post-discharge phone calls were made by 206 students at the university hospital, with an average phone call duration of 18.81 ± 9.79 min.

Ninety-four percent (193/204) of the patients knew their admitting diagnosis and had a follow-up appointment with their PCP. Seventy-two percent (147/204) of patients had a follow-up appointment with a specialist. In 46% (95/204) of patients, a potential barrier to attending the follow-up appointment was identified at the time of the phone call. Of these barriers, 67% (50/74) were due to transportation-related issues. Other barriers were related to insurance/financial issues and logistical issues. Students were able to identify solutions or resources to address the barriers in all instances.

Higher DOT scores were associated with a higher likelihood of good patient-reported outcomes, including patient-reported knowledge of what to do in urgent or emergent situations (OR = 29.52, *p* = 0.002) and a lower likelihood of patients having barriers for attendance of follow-up appointments (OR = 6.08, *p* = 0.006). Most of the comments on lessons learned from the post-discharge phone calls were about issues related to patient follow-up and appointments, medications, patient understanding, and the importance of providing clear instructions.

## DISCUSSION

Medical students are expected to transition to independent discharge communication at the start of residency; therefore, this competency is a high-priority target during undergraduate medical education. The direct observation of students providing discharge instructions to patients revealed that over 75% of students were able to perform these tasks independently in all the domains. All knowledge and skills outcomes improved significantly after the curriculum (*p* < 0.001). The largest improvement in knowledge was in the domain of medication reconciliation. Students were significantly more likely to consider social determinants of health and felt they were substantially better at answering patient questions regarding these social needs after the curriculum. Higher DOT scores were associated with better patient-reported understanding of instructions, adding value by connecting the tool to outcomes that matter to patients.

Communication interventions at the time of discharge have been associated with significantly lower rehospitalization risk, increased adherence to treatment, and higher patient satisfaction.^25,26^ This study responds to the call for interventions to improve communication among care providers, patients, and families and addresses areas of discharge that were identified as important by patients.^27,28^ Our initial patient-reported outcome of improved understanding of discharge instructions may similarly lead to better patient outcomes downstream; this would be an important area for future study.

The DOT builds on the direct observation tool created by Lyons et al. and checklist by Eden et al.^15,16^ By using individual items under each section in the DOT, it helps improve the specificity of the feedback.

Student reflections indicate that the follow-up phone call was a powerful motivator for them to work on improving the discharge instructions they provide to patients. The association of the mean student score on the DOT to patients’ knowledge of what to do if an urgent or emergent problem arose (red flags) is an added strength of this tool. This is important because patients’ knowledge of red flags is one of the pillars that helps prevent readmissions.^29^

These results suggest that the curriculum was highly effective in improving students’ conceptual knowledge, practical awareness, and skills in providing discharge instructions. Given the impact of clear discharge instructions on reducing adverse events, this structured discharge curriculum can be a way to prepare students for this task.

The structured training approach, standardized assessment method with feedback, post-discharge patient phone calls with reflection, and debriefing are the strengths of this curriculum. Having a dedicated faculty member own the delivery and execution of the curriculum and making the completion of direct observation a requirement for completion of the sub-internship are likely why we were able to sustain this intervention.

## LIMITATIONS

Discharge communication is illness-specific, and a student who discharges a patient with a particular illness may not be able to discharge a patient with another illness in the same manner. Our data set did not afford estimating case specificity. We did not use the DOT before implementing the curriculum, so baseline performance is unknown. Thus, we could not measure the extent (if any) of improvement in sub-interns’ ability related to discharge-related tasks after the curriculum. The DOT was completed by residents who were not trained and may have been generous to their students. The DOT scores might have been lower (or different) with trained faculty or other non-trainee observers.

Post-discharge phone calls data was self-reported by students and may be subject to bias, as students may have sought to portray their discharges more favorably. Since this was not counted towards their grade, we believe that students would not intentionally skew the report, but we could not control for this. Tying the DOT scores and phone call data to readmission data would be an ideal end point to determine the effectiveness of this intervention; however, it was beyond the scope of this paper.

## FUTURE DIRECTIONS

This tool has only been used in internal medicine sub-internships and while it may not be generalizable to other specialties or other levels of training, the strategies may be adaptable to other learner groups. While the components of discharging patients are similar in all specialties, the DOT may need some modifications before use in other specialties.

## CONCLUSIONS

This study suggests that structured training in providing discharge instructions improves students’ ability to provide discharge instructions and impacts patient-reported outcomes. This study addresses an important gap and offers valuable preliminary evidence supporting the use of a structured discharge curriculum.

## Supplementary Information

Below is the link to the electronic supplementary material.

Supplementary file1 (DOCX 50.7 KB)

## Data Availability

Data supporting the findings of this study are available from the authors upon reasonable request. All requests will be carefully evaluated on a case‑by‑case basis.
