# Supplementary material for: Aligning Patient Discharge Curricula to Patient-Reported Outcomes in Sub Internship
Source: J Gen Intern Med. 2026 May 11;41(10):2772–8. doi: 10.1007/s11606-026-10495-3 (PMC13421599; doi:10.1007/s11606-026-10495-3)
Supplement: Supplementary file 1 — Supplementary file1 (DOCX 50.7 KB) [file 11606_2026_10495_MOESM1_ESM.docx]

**Appendix 1**

Patient Phone Call script:

General reminders:

PRIOR TO discharging the patient:

● Have all of the discharge procedures completed

● Have Worksheet #1 with you to do the Home-Based Medication Reconciliation.

o Be especially aware of new medications at discharge as well as meds from prior to admit that were discontinued.

● Have Worksheet #3 with you to coordinate follow-up appointments

● Confirm patient phone number for calls

Before the call

● Before the call, discuss with the team any salient issues that should be emphasized during the call: e.g.—new medication changes, instructions, important appointments, etc. Have this as a separate list to remind yourself to address these topics specifically.

● As a reminder, if any discrepancies or issues emerge, please let the patient know you will check with the team, and someone will be contacting them.

● If there is any concern for declining health, discuss with your team to see if the patient needs a sooner urgent care appointment or referral to 911 or ED for immediate medical attention.

● For appointment questions, there are specific clinic numbers on the discharge instructions, but if unable to find them, the general appointment line is 866.600.2273

After the call

● Complete the online form for each completed call: [ Insert Link]

Prior to Discharge (Day of discharge)

. We plan to call you on [ideally 1 day after discharge] once you are home to follow up on how you're doing. Would you be okay with receiving this phone call? [If yes, confirm patient phone number and time to call]

Ask the following questions of the patient prior to discharge:

1. Where (which clinic or health system) do you usually see your doctor? Yes No

2. Tell me about your living situation. Who lives with you? Yes No

3. What type of housing do you live in? Yes No

4. Can you isolate yourself from others in your home? Yes No

5. Do you have a bathroom that others will not use, or do you have a bathroom that can be cleaned each time you use it? Yes No

6. Do you have enough medications to last 14 days? Yes No

7. If a patient is being discharged on oxygen – Do you have any questions about the oxygen? Yes No

8. Do you have access to food and water? Yes No

9. Do you have concerns about transportation needs for upcoming appointments? Yes No

If concerns arise, discuss with your team, and involve discharge planning and social work prior to the patient leaving the hospital.

A. Introduction & Purpose of Phone Call

SubI: Hello Mr./Ms. _____________. I am [caller's name], a medical student from [name of hospital]. You may remember that when you left the [hospital name], I spoke with you briefly about your post-discharge care and I am calling to see how you are doing, if you have any questions and to review your medications.

Can you verify your name and DOB? (If accurate, then continue)

This phone call will likely take us about 15-20 minutes, depending on the number of medications you are taking. Is this a good time to talk?

If yes, continue.

If no, SubI: Is there a better time that I can call you back?

If patient is unable to speak about their medical condition or details of their hospital stay:

SubI: Do you prefer that I speak with a caregiver about your health, and if so, could you identify who this person is?

Can I get your verbal consent to discuss health-related information with that person?

If yes: get name and contact information

SubI: Can you (or a family member) bring all your medications to have them available for this phone call?

At this time, I want to remind you that the information disclosed during this conversation, including anticipatory guidance, does not replace the need to speak with a medical doctor and prompt follow up with your appointments.

B. Health Status Diagnosis EPA 8, EPA 1

SubI: Before you left the hospital, we spoke about your main problem during your hospital stay. This is also called your "primary discharge diagnosis." This can either be a new medical problem or a problem related to your previous health problems. Using your own words, can you explain to me what your main problem or diagnosis during your last hospitalization was? EPA 8

If yes, confirm the patient's knowledge of the discharge diagnosis using the "teach-back" method. After the patient describes their diagnosis, clarify any misconceptions or misunderstandings using a question-and-answer format to keep the patient engaged. ("Post discharge Follow-up Phone Call Script (Patient Version)") ("Post discharge Follow-up Phone Call Script (Patient Version)")

If not, use this opportunity to provide patient education about the discharge diagnosis. Then conduct teach-back to confirm the patient understood.

SubI: Do you have any questions for me about your main problem [diagnosis] or the instructions you were given before you left the hospital? Is there anything I can better explain for you? EPA 8

If yes, explain, using plain language (no jargon or medical terms).

If not, continue.

SubI: Since you left the hospital, how have you been feeling? Do you feel your main problem, [diagnosis], has improved, worsened, or not changed? EPA 1

If improved or no change, continue below.

If patient feels primary condition has worsened or a new problem is mentioned,

● SubI: I'm sorry to hear that. Have you spoken to or seen any doctors or nurses about this since you left the hospital?

● If yes: Who have you spoken with/seen? And what did they suggest you do? Have you done that?

● If no: Please call your MD/seek immediate medical attention depending on how they are feeling. Also, consider letting the team know so that they may reschedule a Discharge Clinic follow up sooner.

● Using clinical judgment, use this conversation to determine if further recommendations, teaching, or interventions are necessary. Discuss this part with the team after the phone call to make sure no further intervention is needed

● Record any action patient/caregiver has taken and your recommendations on the documentation sheet.

SubI: For our patients who have been diagnosed with COVID-19, we ask about several symptoms. I'm going to go through a list with you, please let me know if you have any of these currently.

● Fevers about 100.4F or chills

● Night sweats

● Shortness of breath – difficulty speaking in complete sentences

● Chest pain

● Cough with or without sputum (phlegm) production

● Abdominal pain

● Diarrhea

● Nausea, vomiting

● Generalized weakness

● Muscle aches

● Not able to keep fluids down

For any symptoms that are present, ask if these are stable, improving, or worsening since hospital discharge.

C. Medicines EPA 4

High Alert Medicines

Use the guide below to help monitor medicines with significant risk for adverse events. Have the patient's discharge medication list available for this portion of the conversation.

| Drug Category | What To Look For |
| --- | --- |
| Anticoagulants | Bleeding: if warfarin, then who is managing INR? Do they have an appointment with the anticoagulation clinic (if applicable)? |
| Antibiotics | Emphasize the importance of taking a full antibiotic course despite feeling better. Identify end-date (if known) of medication  Diarrhea; backup method of birth control  Should not be taken at same time as calcium and multivitamin |
| Antiretrovirals | Review profile for drug interactions |
| Insulin | Inquire about fasting blood sugar, hypoglycemic symptoms |
| Antihypertensives | Dizziness  "If yes, suggest patient space out medicines (keep diuretic in a.m.)" ("Post discharge Follow-up Phone Call Script (Patient Version)") |
| Medicines related to primary diagnosis | Focus on acquisition and medication adherence |

SubI: Can I review your hospital discharge medications with you?

For each of the medications the patient talks about, compare it with the discharge Medication list in the following manner:

● Name and strength of medication

● When do you take it? How often do you take it?

● How many tablets do you take at a time?

● Are you experiencing any side effects?

o If yes: let patient know you will get back to them after discussing with your preceptor

● Do you have any other concerns or problems taking this medication?

o If yes, what are the concerns?

SubI: Is there someone who helps you with your medications?

SubI: Do you use a pill box, or do you have a system for remembering when to take medications?

If yes, provide positive reinforcement of using this tool.

If no, suggest using this tool to help remember to take the medicines as ordered. Also suggest taking medications routinely along with other things (for example, putting pillbox next to toothbrush so that they may see them every morning when they brush their teeth, and they may be reminded to take medications)

SubI: What questions do you have today regarding your medicines?

SubI: Does your family or caregiver have any questions or concerns about your medicines?

If so, indicate what the medication list indications show, but also let the team know so that we may be able to clarify any further instructions.

**Please note on the documentation sheet any recommendation you made to the patient and follow-up actions you took. **

SubI: Were there any medications you could not pick up from the pharmacy, or not covered by insurance?

If yes: Do you remember the name of the medication or what it was for?

Inform the team about any medications the patient could not pick up.

D. Clarification of Appointments EPA 8

SubI: I will ensure you and I have the same information about your upcoming appointments and tests. You were given appointments or instructed to call to schedule an appointment. with your doctors [and for lab tests] when you left the hospital. Can you please tell me:

What is the next appointment you have scheduled?

Who is your appointment with?

When is this appointment?

Are you going to be able to make it to your appointment?

● If a patient plans to keep an appointment, ask if you have the phone number to call if something unexpectedly comes up and you can't make the appointment?

● If not, call the general appointment line at 866.600.2273 and they will direct you to the right provider/clinic

● If a patient can't keep an appointment, get the patient to reschedule: As soon as we hang up, can you call to reschedule your appointment?

E. Coordination of Post discharge Home Services (if applicable):EPA 9

SubI: Before you left the hospital, did you know if there was a plan for a visiting nurse at home, homemaker, or respiratory therapist to come visit you? If so, have you been visited by [name of service, e.g., visiting nurse, respiratory therapist] since you came home?

If no, SubI: I will make a note of it and mention it to the team for further follow up

If home equipment was needed--SubI: Have you received the [name of equipment] that was supposed to be delivered?

If no, SubI: I will note it and mention it to the team for further follow up.

F. What to Do If a Problem Arises EPA 8

SubI: Before we hang up, I want to ensure that you know what to do if a medical problem arises. "If you're having an emergency, for example [give disease-specific examples, e.g., chest pain, trouble breathing], what would you do?" ("Post discharge Follow-up Phone Call Script (Patient Version)")

If a patient does not say, "Call 911," explain the need to get an ambulance so he or she can see a doctor immediately and confirm patient understanding.

SubI: And what about if you are having a medical problem that is not an emergency, such as [give disease-specific examples] and want to be seen by your doctor before your next scheduled appointment, what would you do?

"If the patient does not know, tell them: You can call your doctor's office directly and ask for an earlier appointment." ("Post discharge Follow-up Phone Call Script (Patient Version)") The general appointment line is 866.600.2273. Sometimes your doctor is very busy, so if you are having difficulty obtaining an appointment, ask if you can be seen by someone else in the office, such as their colleague, nurse practitioner, or physician's assistant.

Confirm the patient's understanding.

SubI: That's all I needed to talk to you about. We've covered a lot of information. What questions can I answer for you?

If none, SubI: Thank you and have a good day. "If you have to follow up with a patient on anything, remind him or her that you will be calling back." ("Post discharge Follow-up Phone Call Script (Patient Version)")

If the patient has questions, try to answer them to the best of your ability

How much do you agree: This was a helpful educational experience (circle one)

Observer: Strongly disagree 1 2 3 4 5 Strongly agree

Student: Strongly disagree 1 2 3 4 5 Strongly agree

Amount of Time observed: _______ minutes.

From: Re-Engineered Discharge (RED) Toolkit. Post discharge Follow up Phone Call Script (Patient Version)

<https://www.ahrq.gov/patient-safety/settings/hospital/red/toolkit/postdischarge-phone.html>

Accessed Nov 11, 2025

**Appendix 2**

**Discharge Observation Tool**

Using this modified Ottawa scale of 1 to 5 below, please evaluate the student providing

discharge instructions to a patient:

1. Needed complete guidance and was unprepared

2. Able to perform some tasks but required repeated directions

3. Somewhat independent and only needed intermittent assistance

4. Able to do this independently and only needed assistance with nuances or complex situations

5. Able to do this independently and did not need assistance

| 1. Patient Education |  |
| --- | --- |
| a. The student was able to explain major diagnosis & hospital course | 1 2 3 4 5 |
| b. The student was able to explain alarm symptoms or problems requiring return to ER or call to PCP | 1 2 3 4 5 |
| 2. Patient-Centered Care: | 1 2 3 4 5 |
| a. The student was able to elicit concerns about discharge and home support | 1 2 3 4 5 |
| b. The student was able to assess patients' ability to carry out plan (cost, transportation, insurance, etc.) | 1 2 3 4 5 |
| c. The student was able to address the patient's concerns and questions | 1 2 3 4 5 |
| d. The student was able to notify the patient/family of communication with other providers | 1 2 3 4 5 |
| 3. Medication Reconciliation | 1 2 3 4 5 |
| a. The student was able to assess the patient's safety to take meds (eyesight, dexterity, health literacy, cognitive ability) | 1 2 3 4 5 |
| b. The student was able to explain and provide a list of meds to be continued and meds to be STOPPED | 1 2 3 4 5 |
| c. The student was able to explain the indication, instructions, and major side effects of all new medications | 1 2 3 4 5 |
| 4. System-based Practice: (□ Not observed) | 1 2 3 4 5 |
| a. The student was able to involve team members (CM, SW, PT, etc.) in the discharge | 1 2 3 4 5 |
| b. The student was able to communicate a follow-up plan with other MDs | 1 2 3 4 5 |
| c. The student was able to explain the timeframe of future tests that require follow-up (i.e., labs, etc.), including which clinician will follow | 1 2 3 4 5 |
| 5. Communication Skills: | 1 2 3 4 5 |
| a. 4, The student was able to use appropriate language and terminology (avoidance of medical jargon) | 1 2 3 4 5 |
| b. The student was able to use the teach-back method during the discussion with the patient | 1 2 3 4 5 |
| c. The student was able to answer questions appropriately, demonstrate professionalism/humanism, and use appropriate non-verbal communication | 1 2 3 4 5 |

**Appendix 3:**

**Content Validity: Relevance Ratings of DOT items**

|  | Overall Relevance  Mean ± Std. Dev (n = 31) |
| --- | --- |
| 1. Patient Education |  |
| a. The student was able to explain major diagnosis & hospital course | 3.77 ± 0.43 |
| b. The student was able to explain alarm symptoms or problems requiring return to ER or call to PCP | 3.90 ± 0.3 |
| 2. Patient-Centered Care: |  |
| a. The student was able to elicit concerns about discharge and home support | 3.61± 0.56 |
| b. The student was able to assess patients' ability to carry out plan (cost, transportation, insurance, etc.) | 3.23 ± 0.88 |
| c. The student was able to address the patient's concerns and questions | 3.58 ± 0.62 |
| d. The student was able to notify the patient/family of communication with other providers | 2.84 ± 0.73 |
| 3. Medication Reconciliation |  |
| a. The student was able to assess the patient's safety to take meds (eyesight, dexterity, health literacy, cognitive ability) | 3.41± 0.67 |
| b. The student was able to explain and provide a list of meds to be continued and meds to be STOPPED | 3.90 ± 0.30 |
| c. The student was able to explain the indication, instructions, and major side effects of all new medications | 3.33 ± 0.71 |
| 4. System-based Practice: |  |
| a. The student was able to involve team members (CM, SW, PT^,^ etc.) in the discharge | 3.06 ± 0.77 |
| b. The student was able to communicate a follow-up plan with other MDs | 3.41 ± 0.72 |
| c. The student was able to explain the timeframe of future tests that require follow-up (i.e., labs, etc.), including which clinician will follow | 3.52 ± 0.63 |
| 5. Communication Skills: |  |
| a. 4, The student was able to use appropriate language and terminology (avoidance of medical jargon) | 3.84 ±0.37 |
| b. The student was able to use the teach-back method during the discussion with the patient | 3.52 ±0.63 |
| c. The student was able to answer questions appropriately, demonstrate professionalism/humanism, and use appropriate non-verbal communication | 3.57 ±0.57 |

^Rating Scale: 1 not relevant, 2 somewhat relevant, 3 quite relevant, 4 highly relevant in the assessment of discharge instructions provided by students to patients^

^ER -Emergency Room, PCP- Primary Care Provider, CM Case Manager, SW Social Worker, PT Physical Therapist^

**Appendix 4.**

Psychometric Characteristics of the Discharge Tool: Rater Analysis and Item Characteristics

| Item | Rater Consistency | | | Rater Accuracy | | | Item Characteristics | |
| --- | --- | --- | --- | --- | --- | --- | --- | --- |
|  | ICC | 95% CI | | ICC | 95% CI | | Item Discrimination | Inter-Item Correlation |
| 1 | 0.76 | 0.54 | 0.95 | 0.74 | 0.57 | 0.91 | 0.87 | 0.76 |
| 2 | 0.51 | 0.27 | 0.87 | 0.53 | 0.38 | 0.69 | 0.81 | 0.77 |
| 3 | 0.60 | 0.35 | 0.90 | 0.68 | 0.51 | 0.86 | 0.92 | 0.76 |
| 4 | 0.42 | 0.20 | 0.82 | 0.54 | 0.38 | 0.70 | 0.83 | 0.77 |
| 5 | 0.79 | 0.58 | 0.96 | 0.81 | 0.63 | 0.99 | 0.92 | 0.75 |
| 6 | 0.57 | 0.32 | 0.89 | 0.64 | 0.48 | 0.81 | 0.83 | 0.76 |
| 7 | 0.37 | 0.16 | 0.79 | 0.39 | 0.26 | 0.52 | 0.74 | 0.78 |
| 8 | 0.83 | 0.65 | 0.97 | 0.86 | 0.67 | 1.05 | 0.90 | 0.76 |
| 9 | 0.48 | 0.24 | 0.85 | 0.54 | 0.39 | 0.69 | 0.82 | 0.77 |
| 10 | 0.59 | 0.34 | 0.90 | 0.62 | 0.46 | 0.78 | 0.80 | 0.77 |
| 11 | 0.43 | 0.21 | 0.83 | 0.57 | 0.40 | 0.75 | 0.78 | 0.77 |
| 12 | 0.56 | 0.31 | 0.89 | 0.59 | 0.44 | 0.75 | 0.92 | 0.75 |
| 13 | 0.70 | 0.46 | 0.94 | 0.69 | 0.53 | 0.86 | 0.90 | 0.76 |
| 14 | 0.82 | 0.63 | 0.97 | 0.85 | 0.65 | 1.04 | 0.93 | 0.75 |
| 15 | 0.66 | 0.42 | 0.92 | 0.69 | 0.52 | 0.86 | 0.90 | 0.76 |
| 16 | 0.67 | 0.42 | 0.93 | 0.75 | 0.57 | 0.93 | 0.94 | 0.75 |
| Overall | 0.80 | 0.59 | 0.96 | 0.72 | 0.56 | 0.88 | 0.86 | 0.76 |

**Appendix 5**

Pre-Curriculum Survey

As part of your Internal Medicine Sub-Internship, we aim to improve your readiness to be an integral part of patient handoffs and care transitions.  This pre-curriculum survey should take approximately 10 minutes to complete, and will help us understand how to best prepare you in patient discharge practices for your intern year.

Please do not hesitate to contact us with any questions, suggestions, or comments.

What is your hospital site?

I have previously been involved with a patient discharge from the hospital

Yes

No

I have previously received training in patient discharges

Yes

No

The questions below pertain to your awareness of the impact of social determinants of health on post discharge care

How aware are you of the degree of impact of housing instability on the post discharge care of our patients?

1. Not at all aware
2. Slightly aware
3. Somewhat aware
4. Moderately aware
5. Extremely aware

How aware are you of the degree of impact of food insecurity on the post discharge care of our patients?

1. Not at all aware
2. Slightly aware
3. Somewhat aware
4. Moderately aware
5. Extremely aware

How aware are you of the degree of impact of access to utilities (water, electricity, heat) on the post discharge care of our patients?

1. Not at all aware
2. Slightly aware
3. Somewhat aware
4. Moderately aware
5. Extremely aware

How aware are you of the degree of impact of access to transportation on the post discharge care of our patients?

1. Not at all aware
2. Slightly aware
3. Somewhat aware
4. Moderately aware
5. Extremely aware

How aware are you of the degree of impact of interpersonal safety on the post discharge care of our patients?

1. Not at all aware
2. Slightly aware
3. Somewhat aware
4. Moderately aware
5. Extremely aware

Please list the top three interventions you took to ensure that there is good communication between you and the patient before you start discussing discharge instructions

Please list the top three elements that you addressed when providing discharge education to a patient

The questions below pertain to key elements of the discharge process:

I know how to do medication reconciliation

1. Strongly disagree
2. Somewhat disagree
3. Neither agree nor disagree
4. Agree
5. Strongly agree

I know how to write a discharge summary

1. Strongly disagree
2. Somewhat disagree
3. Neither agree nor disagree
4. Agree
5. Strongly agree

I know the roles of a discharge planner, social worker, and case manager

1. Strongly disagree
2. Somewhat disagree
3. Neither agree nor disagree
4. Agree
5. Strongly agree

The questions below pertain to discharge education:

I provide discharge education to my patients

1. None of the time
2. Some of the time
3. Most of the time
4. All of the time

When I provide discharge education to my patients:

I consider transportation needs

1. Some of the time
2. Most of the time
3. All the time

I consider insurance

1. Some of the time
2. Most of the time
3. All the time

I consider financial concerns

1. Some of the time
2. Most of the time
3. All the time

I consider home needs (O2, RN, PT, etc)

1. Some of the time
2. Most of the time
3. All the time

The questions below pertain to your ability to address the social determinants of health that can impact post discharge care

How would you describe your ability to address patients’ concerns and answer questions about housing instability?

1. I need complete guidance
2. I require assistance most of the time
3. I need intermittent assistance
4. I need assistance only with complex situations
5. I do not need assistance

How would you describe your ability to address patients’ concerns and answer questions about food insecurity?

1. I need complete guidance
2. I require assistance most of the time
3. I need intermittent assistance
4. I need assistance only with complex situations
5. I do not need assistance

How would you describe your ability to address patients’ concerns and answer questions about access to utilities (water, electricity, heat)?

1. I need complete guidance
2. I require assistance most of the time
3. I need intermittent assistance
4. I need assistance only with complex situations
5. I do not need assistance

How would you describe your ability to address patients’ concerns and answer questions about access to transportation?

1. I need complete guidance
2. I require assistance most of the time
3. I need intermittent assistance
4. I need assistance only with complex situations
5. I do not need assistance

How would you describe your ability to address patients’ concerns and answer questions about interpersonal safety?

1. I need complete guidance
2. I require assistance most of the time
3. I need intermittent assistance
4. I need assistance only with complex situations
5. I do not need assistance

Please list AT LEAST 3 things you would like to learn about discharge practices during this rotation.

Thank you for taking the time to complete this survey.  We appreciate all of your hard work!

**Appendix 6**

Post-Curriculum Survey

As we near the end of your Internal Medicine Sub-Internship, we hope you feel more confident with patient handoffs and care transitions.  This post-curriculum survey should take approximately 10 minutes to complete.  Thanks for your time, and we wish you the best as you move forward with your M4 year!

Please do not hesitate to contact us with any questions, suggestions, or comments.

What is your hospital site?

The questions below pertain to your awareness of the impact of social determinants of health on post discharge care

How aware are you of the degree of impact of housing instability on the post discharge care of our patients?

1. Not at all aware
2. Slightly aware
3. Somewhat aware
4. Moderately aware
5. Extremely aware

How aware are you of the degree of impact of food insecurity on the post discharge care of our patients?

1. Not at all aware
2. Slightly aware
3. Somewhat aware
4. Moderately aware
5. Extremely aware

How aware are you of the degree of impact of access to utilities (water, electricity, heat) on the post discharge care of our patients?

1. Not at all aware
2. Slightly aware
3. Somewhat aware
4. Moderately aware
5. Extremely aware

How aware are you of the degree of impact of access to transportation on the post discharge care of our patients?

1. Not at all aware
2. Slightly aware
3. Somewhat aware
4. Moderately aware
5. Extremely aware

How aware are you of the degree of impact of interpersonal safety on the post discharge care of our patients?

1. Not at all aware
2. Slightly aware
3. Somewhat aware
4. Moderately aware
5. Extremely aware

Please list the top three interventions you took to ensure that there is good communication between you and the patient before you start discussing discharge instructions

Please list the top three elements that you addressed when providing discharge education to a patient

The questions below pertain to key elements of the discharge process:

I know how to do medication reconciliation

1. Strongly disagree
2. Somewhat disagree
3. Neither agree nor disagree
4. Agree
5. Strongly agree

I know how to write a discharge summary

1. Strongly disagree
2. Somewhat disagree
3. Neither agree nor disagree
4. Agree
5. Strongly agree

I know the roles of a discharge planner, social worker, and case manager

1. Strongly disagree
2. Somewhat disagree
3. Neither agree nor disagree
4. Agree
5. Strongly agree

The questions below pertain to discharge education:

I provide discharge education to my patients

1. None of the time
2. Some of the time
3. Most of the time
4. All of the time

When I provide discharge education to my patients:

I consider transportation needs

1. Some of the time
2. Most of the time
3. All the time

I consider insurance

1. Some of the time
2. Most of the time
3. All the time

I consider financial concerns

1. Some of the time
2. Most of the time
3. All the time

I consider home needs (O2, RN, PT, etc)

1. Some of the time
2. Most of the time
3. All the time

The questions below pertain to your ability to address the social determinants of health that can impact post discharge care

How would you describe your ability to address patients’ concerns and answer questions about housing instability?

1. I need complete guidance
2. I require assistance most of the time
3. I need intermittent assistance
4. I need assistance only with complex situations
5. I do not need assistance

How would you describe your ability to address patients’ concerns and answer questions about food insecurity?

1. I need complete guidance
2. I require assistance most of the time
3. I need intermittent assistance
4. I need assistance only with complex situations
5. I do not need assistance

How would you describe your ability to address patients’ concerns and answer questions about access to utilities (water, electricity, heat)?

1. I need complete guidance
2. I require assistance most of the time
3. I need intermittent assistance
4. I need assistance only with complex situations
5. I do not need assistance

How would you describe your ability to address patients’ concerns and answer questions about access to transportation?

1. I need complete guidance
2. I require assistance most of the time
3. I need intermittent assistance
4. I need assistance only with complex situations
5. I do not need assistance

How would you describe your ability to address patients’ concerns and answer questions about interpersonal safety?

1. I need complete guidance
2. I require assistance most of the time
3. I need intermittent assistance
4. I need assistance only with complex situations
5. I do not need assistance

Any remaining questions you have about the discharge process or suggestions for how we can improve this curriculum?

Thank you for taking the time to complete this survey.  We appreciate all of your hard work!
